# Spatiotemporal Optimization for the Placement of Automated External Defibrillators Using Mobile Phone Data

**DOI:** 10.3390/ijgi12030091

**Published:** 2023-02-23

**Authors:** Jielu Zhang, Lan Mu, Donglan Zhang, Janani Rajbhandari-Thapa, Zhuo Chen, José A. Pagán, Yan Li, Heejung Son, Junxiu Liu

**Affiliations:** 1 Department of Geography, University of Georgia, Athens, GA 30602, USA; 2 Division of Health Services Research, Department of Foundations of Medicine, New York University Long Island School of Medicine, New York, NY 11501, USA; 3 Department of Health Policy and Management, College of Public Health, University of Georgia, Athens, GA 30602, USA; 4 School of Economics, University of Nottingham Ningbo China, Ningbo 315100, China; 5 Department of Public Health Policy and Management, School of Global Public Health, New York University, New York, NY 10003, USA; 6 Department of Population Health Science and Policy, Icahn School of Medicine at Mount Sinai, New York, NY 10029, USA; 7 Department of Obstetrics, Gynecology, and Reproductive Science, Icahn School of Medicine at Mount Sinai, New York, NY 10029, USA; 8 Department of Epidemiology & Biostatistics, College of Public Health, University of Georgia, Athens, GA 30602, USA

**Keywords:** optimization placement, automated external defibrillator, out-of-hospital cardiac arrest, maximal covering location problem, overlayed spatio-temporal optimization

## Abstract

With over 350,000 cases occurring each year, out-of-hospital cardiac arrest (OHCA) remains a severe public health concern in the United States. The correct and timely use of automated external defibrillators (AEDs) has been widely acknowledged as an effective measure to improve the survival rate of OHCA. While general guidelines have been provided by the American Heart Association (AHA) for AED deployment, the lack of detailed instructions hindered the adoption of such guidelines under dynamic scenarios with various time and space distributions. Formulating the AED deployment as a location optimization problem under budget and resource constraints, we proposed an overlayed spatio-temporal optimization (OSTO) method, which accounted for the spatiotemporal heterogeneity of potential OHCAs. To highlight the effectiveness of the proposed model, we applied the proposed method to Washington DC using user-generated anonymized mobile device location data. The results demonstrated that optimization-based planning provided an improved AED coverage level. We further evaluated the effectiveness of adding additional AEDs by analyzing the cost-coverage increment curve. In general, our framework provides a systematic approach for municipalities to integrate inclusive planning and budget-limited efficiency into their final decision-making. Given the high practicality and adaptability of the framework, the OSTO is highly amenable to different healthcare facilities’ deployment tasks with flexible demand and resource restraints.

## 1. Introduction

Out-of-hospital cardiac arrest (OHCA) is the loss of functional cardiac mechanical activity associated with an absence of systemic circulation occurring outside of a hospital setting [1]. More than 350,000 people suffer from OHCA in the United States (US) each year [2], and less than 6% of them survive [3]. From collapse to treatment, every additional minute will reduce the probability of survival by up to 10% [4]. Public access defibrillation programs using automated external defibrillators (AEDs) are practical and have been linked to a significant increase in the OHCA survival rate [5–7]. However, fewer than 12% of individuals with OHCAs have an AED applied before the emergency medical services (EMSs) arrive [1]. Several factors responsible for this rate include an unawareness of AED locations, a lack of AED training, bystander apathy, and concerns about liability [8–10]. Another major factor is the accessibility to AEDs [11–13]. General guidelines proposed by the American Heart Association (AHA) [14] suggested that AEDs should be deployed in areas at risk of cardiac arrest, which did not provide a clear plan for delimiting risk boundaries and positioning AEDs. Thus, determining the areas of potential OHCAs is the first step before optimizing the AED placement.

Many previous studies predicted the potential OHCAs using population distribution or historical OHCAs [15–17]. However, the bias of using population distribution to model real-time spatial population dynamics has long been revealed [15]. On the other hand, due to practical factors, the historical OHCA data are not accessible in many scenarios. To our knowledge, the overwhelming majority of research on AED placement is limited to western countries with well-developed systematic programs and dedicated personnel for collecting incident locations of OHCAs, including the National Emergency Medical Services Information System in the US, the Resuscitation Outcomes Consortium in North America, and Scottish Ambulance Service in Scotland, among others. These programs are not always available in some underdeveloped countries or regions due to the global disparity and imbalance in the development of healthcare systems. Additionally, for some small areas, it is impractical to collect sufficient historical OHCA data to perform effective AED placement optimization. In Washington DC, for example, the total number of cardiac arrests was 605 in 2019, and the number of estimated OHCA cases was only 179 [18,19]. We overcome the drawbacks of the aforementioned methods by representing the potential distribution of OHCAs with dynamic point of interest (POI) [20] visit data collected from mobile devices.

In contrast to historical OHCA data, which are usually sparse and uneven across nations, mobile location data can be acquired in most countries [20] owing to the extensive reach of mobile phones. By utilizing such data, we may overcome the intrinsic hindrance in gathering OHCA cases and formulate a method that has the potential to span both developed and developing regions, bridging the existing public health disparities between them. POI visit data represent real-time population movement, effectively reflecting potential OHCA risk areas across space and time. Based on the statistics on cardiac arrest location types in the US [18], 60.32% OHCAs occurred in public or commercial buildings. Applying the North American Industry Classification System (NAICS) [21] on POI visit data suggested that almost all the POIs are subordinate to the public and commercial buildings where most OHCAs occurred.

In addition, the majority of the studies and AHA guidelines for AED placement focus on accessibility concerning the spatial pattern of potential OHCAs, including using the incident location type to predict potential locations at risk of cardiac arrest [22,23], leveraging GIS techniques [24], or optimization algorithm [15,25,26] to improve the access level of AEDs and delivering AEDs with the aid of drones [16,27]. Although improvements have been made by integrating spatial patterns into AED deployment plans, temporal access has been largely ignored in previous research. Given that there exists a substantial difference in the occurrence and survival of OHCAs between the different times of day and different days of the week [28,29], a spatio-temporal analysis method is needed to better model the spatio-temporal characteristics of OHCAs.

In this study, we propose an adaptable overlayed spatio-temporal optimization (OSTO) method to optimize the AED placement using fine-grained analysis in time and space. Taking Washington DC as a case study, the results demonstrate that our proposed methods can successfully maximize the AED performance with respect to all time periods as well as improve the AED access level. We also evaluated the cost′coverage increment curve to determine the most suitable number of total deployed AEDs considering the AED coverage efficiency and financial budget. Our method presents a general framework that can be practically adapted to other facility deployment optimization planning when both spatial and temporal factors are important.

## 2. Overlayed Spatio-Temporal Optimization Method

**Figure 1.**
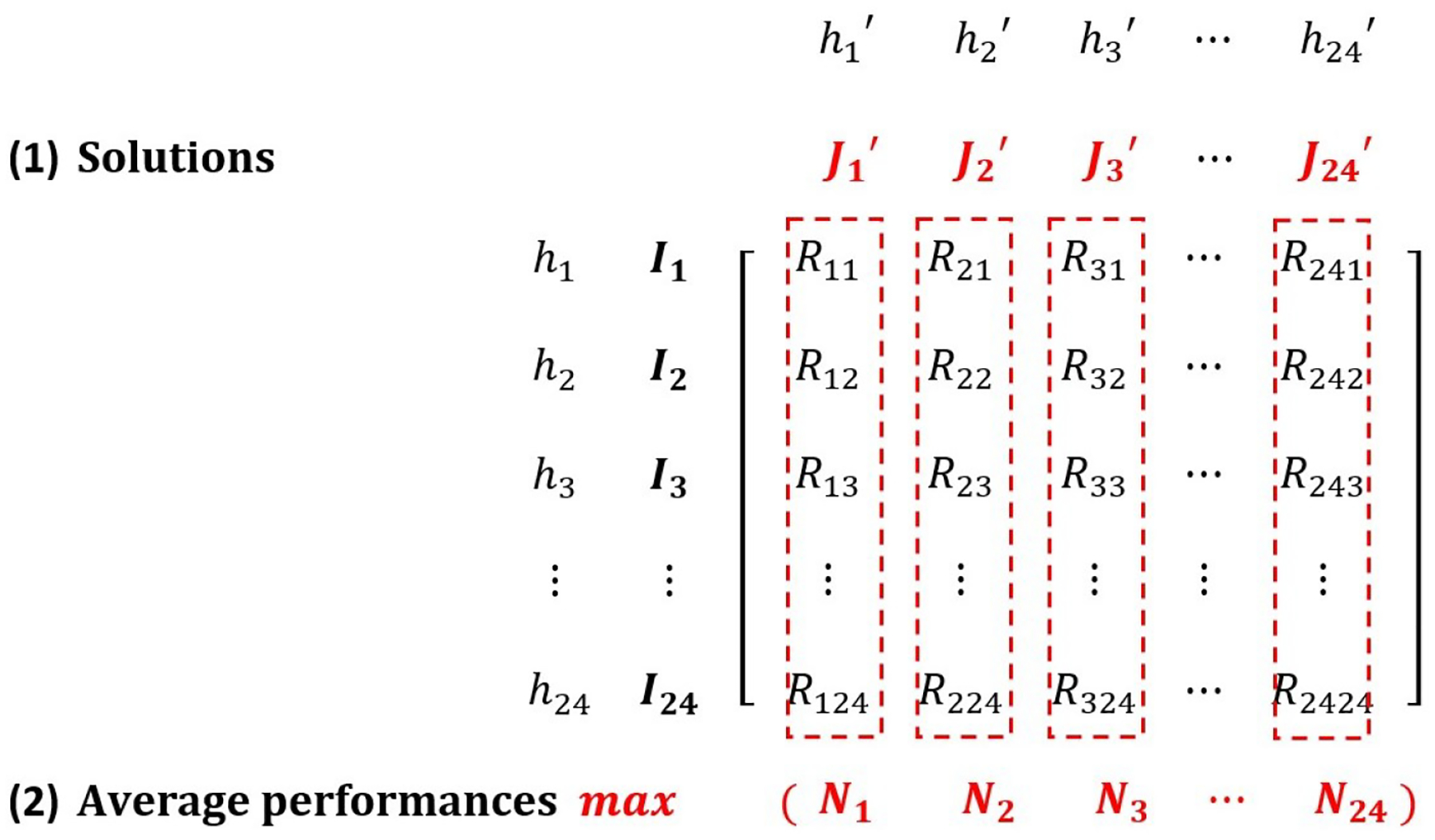
A Matrix Describing the Process of the Overlayed Spatio-Temporal Optimization. Both $h_{1},h_{2},h_{3},\;\ldots \;,h_{24}$ and $h_{1}^{\prime },h_{2}^{\prime },h_{3}^{\prime },\;\ldots \;,h_{24}^{\prime }$ refer to 24 h a day. $I_{1},I_{2},I_{3},\;\ldots \;,I_{24}$ indicate 24 hourly sets of POI visitor distribution. $J_{1}^{\prime },J_{2}^{\prime },J_{3}^{\prime },\;\ldots \;,J_{24}^{\prime }$ represents 24 hourly AED solutions computed based on the visitor distribution of the hour itself. $R_{hh^{\prime }}\left(h,h^{\prime }\in 1,2,3,\;\ldots \;,24\right)$ means the optimized AED coverage rate applying the $h^{\prime }$th solution on hth set of POI visitor distribution. $N_{1},N_{2},N_{3},\;\ldots \;,N_{24}$ means the 24 average performances for each solution. For instance, $N_{1}$ is averaged over $R_{11},R_{12},R_{13},\;\ldots \;R_{124}$, and $N_{2},N_{3},\;\ldots \;,N_{24}$ is calculated similarly.

The proposed OSTO method includes two stages (Figure 1). The first stage aims to maximize the AED coverage rate by hour and obtain a total of 24 solutions $\left\{J_{1}^{\prime },J_{2}^{\prime },J_{3}^{\prime },\;\ldots \;,J_{24}^{\prime }\right\}$ corresponding to 24 h, with each solution representing a spatial distribution of optimized AEDs. For each hour, the optimized AED coverage rate $\left\{R_{11},R_{22},R_{33},\;\ldots \;,R_{2424}\right\}$ was calculated using the hourly solution to cover the POI visitor distribution of the hour itself. The second stage is to apply each solution into 24 sets of visitor distribution corresponding to 24 h $\left\{I_{1},I_{2},I_{3},\;\ldots \;,I_{24}\right\}$, calculate the average performance $\left\{N_{1},N_{2},N_{3},\;\ldots \;,N_{24}\right\}$ of each solution over 24 h, and identify the best solution out of the 24 sets. The next two subsections will illustrate details about the calculation process for these two stages.

### 2.1. Optimizing AED Placement by Hour

This section aims to illustrate the first stage of the OSTO. The mixed-integer programming (MIP) model based on a branch-and-bound algorithm [30] was deployed to solve the maximal covering location problem (MCLP), which is to locate a restrained number of facilities to maximize the population covered within a particular service distance [31]. Our scenario of optimizing the AED placement aimed to identify a set of locations where placing AEDs would maximize the number of covered POI visitors. The objective function (1) and four constraints (2)–(5) to the MCLP for AED optimizing placement can be expressed as follows:

(1) $$\text{Maximize }z_{h}=\sum_{i\in I_{h}}a_{i}y_{i}\text{ for all }h\in H$$

(2) $$\text{Subject to }\sum_{j\in N_{i}}x_{j}⩾y_{i}\text{ for all }i\in I_{h}$$

(3) $$\sum_{j\in J}x_{j}=k$$

(4) $$x_{j}=0\text{ or }1\text{ for all }j\in J$$

(5) $$y_{i}=0\text{ or }1\text{ for all }i\in I_{h}$$

where:

h = denotes each hour;

H = denotes the set of 24 h 1, … ,h… ,24;

i = denotes each POI site;

$I_{h}$ = denotes the set of POI sites for each hour;

j = denotes each AED candidate site;

J = denotes the set of AED candidate sites;

$a_{i}$ = the number of visitors to be served at POI site i;

k = the number of AEDs to be located;

$d_{ij}$ = the shortest distance from site i to site j; S = the distance beyond which a POI site is considered uncovered;

$N_{i}=\left\{j\in J|d_{ij}⩽S\right\}$, denotes the set of AED candidate sites that can cover visitors in the POI site I;

$Z_{h}$ = denotes the number of covered POI visitors;

$x_{j}=\{\begin{array}{ll} 1 & \text{if an }AED\text{ is allocated to site }j\\ 0 & \text{else } \end{array}$ $y_{i}=\{\begin{array}{ll} 1 & \text{if one or more AED candidate sites are established at sites in the set }N_{i}\\ 0 & \text{else } \end{array}$

The constraints can be explained as follows: for AED selection (4), if an AED is allocated to location j, the $x_{j}$ is assigned 1, otherwise the $x_{j}$ is assigned 0. For budget constraint (3), the total number of AEDs to be located is k. For POI sites (2) and (5), if there exists at least one established AED from which a POI site i is within the distance of S, then $y_{i}$ is assigned 1; otherwise $y_{i}$ is assigned 0. Embedding these constraints and the objective function into the MIP model, we conducted the model by the hour, and 24 solutions were computed. The potential AED locations provided for the MIP model to select the optimized AED locations are named AED candidate sites in this study.

### 2.2. Identifying the Final Solution of Optimized AEDs

This section aimed to illustrate the second stage of the OSTO. The solution of optimized AED locations at each specific hour h was then applied 24 times to hourly visitor distribution, and then the average performance of each solution over 24 sets of POI distribution was calculated $\left(N_{J_{h^{\prime }}^{\prime }}\right)$. A total of $24N_{J_{h^{\prime }}^{\prime }}\text{s}$ were generated and the final solution was identified as the solution with maximal $N_{J_{h^{\prime }}^{\prime }}$.

Calculation process for identifying the final solution:

(6) $$O_{i}=\{\begin{array}{ll} 1 & \text{min}\left(d_{J_{h^{\prime }}^{\prime }i}\right)⩽S\\ 0 & \text{else} \end{array}\;i\in I_{h};j^{\prime }\in J_{h^{\prime }}^{\prime };h^{\prime }\in H$$

(7) $$R_{J_{h^{\prime }}^{\prime }I_{h}}=\frac{\sum_{i\in I_{h}}O_{i}a_{i}}{\sum_{i\in I_{h}}a_{i}}\;h\in H;h^{\prime }\in H$$

(8) $$N_{J_{h^{\prime }}^{\prime }}=\text{Avg}\left(R_{J_{h^{\prime }}I_{1}},R_{J_{h^{\prime }}I_{2}},R_{J_{h^{\prime }}I_{3}},\;\ldots ,R_{J_{h^{\prime }}I_{24}}\right)\;h^{\prime }\in H$$

(9) $$\text{Objective Max}\left(N_{J_{1}^{\prime }},N_{J_{2}^{\prime }},N_{J_{3}^{\prime }},\;\ldots ,N_{J_{24}^{\prime }}\right)$$

where:

$h^{\prime }$ = also denotes each hour, used for distinguishing itself from h;

H = denotes the set of 24 h 1, … ,h… ,24;

i = denotes each POI site;

$I_{h}$ = denotes the set of POI sites for each hour;

$j^{\prime }$ = denotes each optimized AED;

$J_{h^{\prime }}^{\prime }$ = denotes the set of optimized AEDs for each hour;

$\text{Min}\left(d_{J_{h^{\prime }}^{\prime }i}\right)$ = denotes the minimum distance between each site in the set of optimized AEDs and a POI site;

S = the distance beyond which a POI site is considered uncovered;

$O_{i}$ = denotes whether a POI site i is covered by a set of optimized AEDs;

$a_{i}$ = the number of visitors to be served at POI site i;

$R_{J_{h^{\prime }}^{\prime }I_{h}}$ = denotes the optimized AED coverage rate for each hour $h^{\prime }$;

$N_{J_{h^{\prime }}^{\prime }}^{h^{\prime }}$ = denotes the average performance of each hour’ s set of optimized AEDs.

Four steps for identifying the final solution are: (6) Calculate whether a POI site i is covered by any site of an AED solution $\left(O_{i}\right)$; (7) Obtain the optimized AED coverage rate $\left(R_{J_{h^{\prime }}^{\prime }}I_{h}\right)$ using a solution in a specific hour $\left(h^{\prime }\right)$ to cover a POI visitor distribution in another specific hour (h); (8) Compute the averaged performance $\left(N_{J_{h^{\prime }}^{\prime }}\right)$ of a solution in a specific hour $\left(h^{\prime }\right)$; (9) Compare the average performances between 24 h and identify the maximal one $\left(\text{Max}\left(N_{J_{h^{\prime }}^{\prime }}\right)\right)$.

### 2.3. Cost-Coverage Increment Analysis

The cost-coverage increment curve was evaluated by assessing the increased average performance obtained from an increasing number of AEDs. Specifically, it is calculated by dividing the increased AED average performance $\left(\Delta N_{J_{h^{\prime }}^{\prime }}\right)$ by the increased number of AEDs to be located (Δk) (the intervention cost). Steps for calculating the average performance of a certain k number of AEDs were as follows: (1) The optimized AED locations are computed based on the visitor distribution of the hour $\left(\hat{h}\right)$ with the highest performance; (2) Calculate the average performance $\left(N_{J_{h^{\prime }}^{\prime }}\right)$ of the set of optimized AED locations on all 24 h visitor distributions; (3) The final cost-coverage increment curve is generated based on $\Delta N_{J_{h^{\prime }}^{\prime }}/\Delta k$.

## 3. Application in Washington DC

### 3.1. Data Source and Preprocessing

#### 3.1.1. POI Visit Data

**Figure 2.**
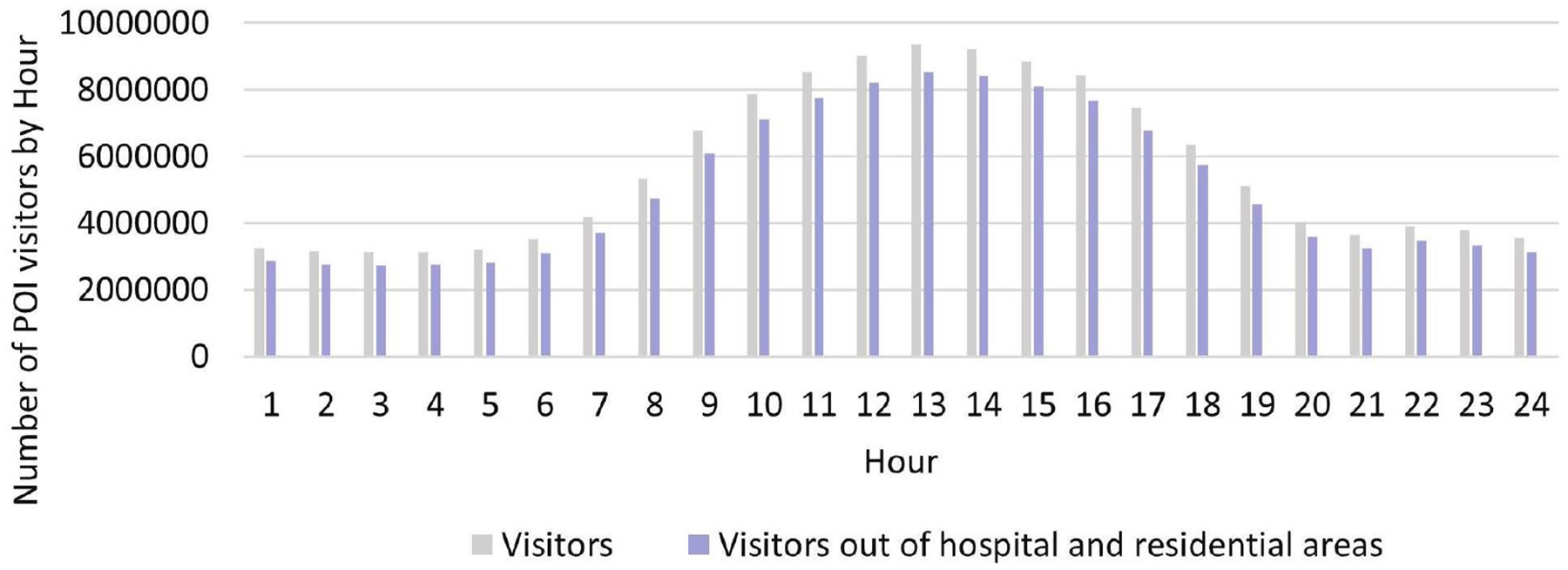
Number of POI visitors by Hour in Washington DC.

The POI visit count data in 2019 in Washington DC originates from the SafeGraph Pattern Dataset [20], indicating that the count of visits to each POI during each hour accumulated across the whole year of 2019. Considering the fact that the visit duration must last at least 4 min to count as valid for a given POI and that the visitor would be counted once in each hour if they stay for multiple hours [20], visit counts and visitors are interchangeable in our study. Since we focused on deploying public-access defibrillators, we excluded residential areas that are not publicly accessible. Considering the full equipment with healthcare facilities including AEDs in hospitals, we also excluded hospital areas. After removing the POI visit counts in residentials and hospitals, 134,850,972 POI total visit counts were used for analysis. The number of visitors per hour is shown in Figure 2. Considering the computational efficiency, we randomly sampled 1/6 visitor locations per day for the optimization analysis. The sample size was $N_{\text{sample}}=N_{\text{total}}/365/6=61,575$. The value calculated from $N_{\text{total}}/365$ means the average daily visits, and further dividing by six means that one in every six POI visit counts.

#### 3.1.2. Existing AEDs and AED Candidate Sites

The data of 2302 AEDs in Washington DC were obtained from Open Data DC (updated until 8 December 2021) [32]. A total of 2113 AEDs were left after removing the existing AEDs in hospitals and residential areas. A total of 18,098 AED candidate sites were generated from the centroids of buildings other than hospitals and residential areas, and building data were obtained from the Open Street Map [33].

#### 3.1.3. Hospital and Residential Data

Data on 54 hospitals were accessed from the Geographic Names Information System (GNIS) (updated until 25 August 2021) [34]; the land use data with 137,626 polygons came from Open Data DC (updated until 18 March 2022), which includes residential data [32].

### 3.2. Analysis

In our model, we set k as the maximum number of locations where AEDs could be deployed. We set k as equal to 100, 200, and 300 separately to explore the optimization under different situations. The distance S beyond which a POI site is considered uncovered is set as 100 m as suggested by the AHA [5]. To simulate the mobility of visitors, we randomly spread out the visitors of each POI in each hour into a 5 min walking circle. Thus, the $a_{i}$ (the number of POI visitors to be served at site i) that was used to maximize the $z_{h}$ (the number of covered POI visitors) in the maximizing function $\left(\text{Maximize }z_{h}=\sum_{i\in I_{h}}a_{i}y_{i}\right)$ was set to 1. Hence, the $z_{h}$ value is only determined by $y_{i}$ (whether the visitor is covered or not). The radius of the walking circle is calculated as 414.3 m based on the average comfortable gait speed (138.10 cm/s) of all the adults of different ages [35]. For each k value, we conducted the first stage of the OSTO, and then compared the trendlines of the AED coverage rate across 24 h between different ks. Since trends were similar between different ks, we conducted the second stage of the OSTO only with k equals to 100. The trend of the average performance $\left(N_{J_{h^{\prime }}^{\prime }}\right)$ of each solution across 24 h was then calculated. We used the Python (Python 3.7.11) programming language to code the algebraic formulation of the model and used the Gurobi [30] solver to solve the optimization problem. The whole process took 446,293.68 s of computing time with the use of a workstation with Intel^®^ Xeon^®^ CPU E5-1650 6 cores 3.78-GHz processor and 128 GB of RAM.

### 3.3. Results

#### 3.3.1. Applying the OSTO in Washington DC

**Figure 3.**
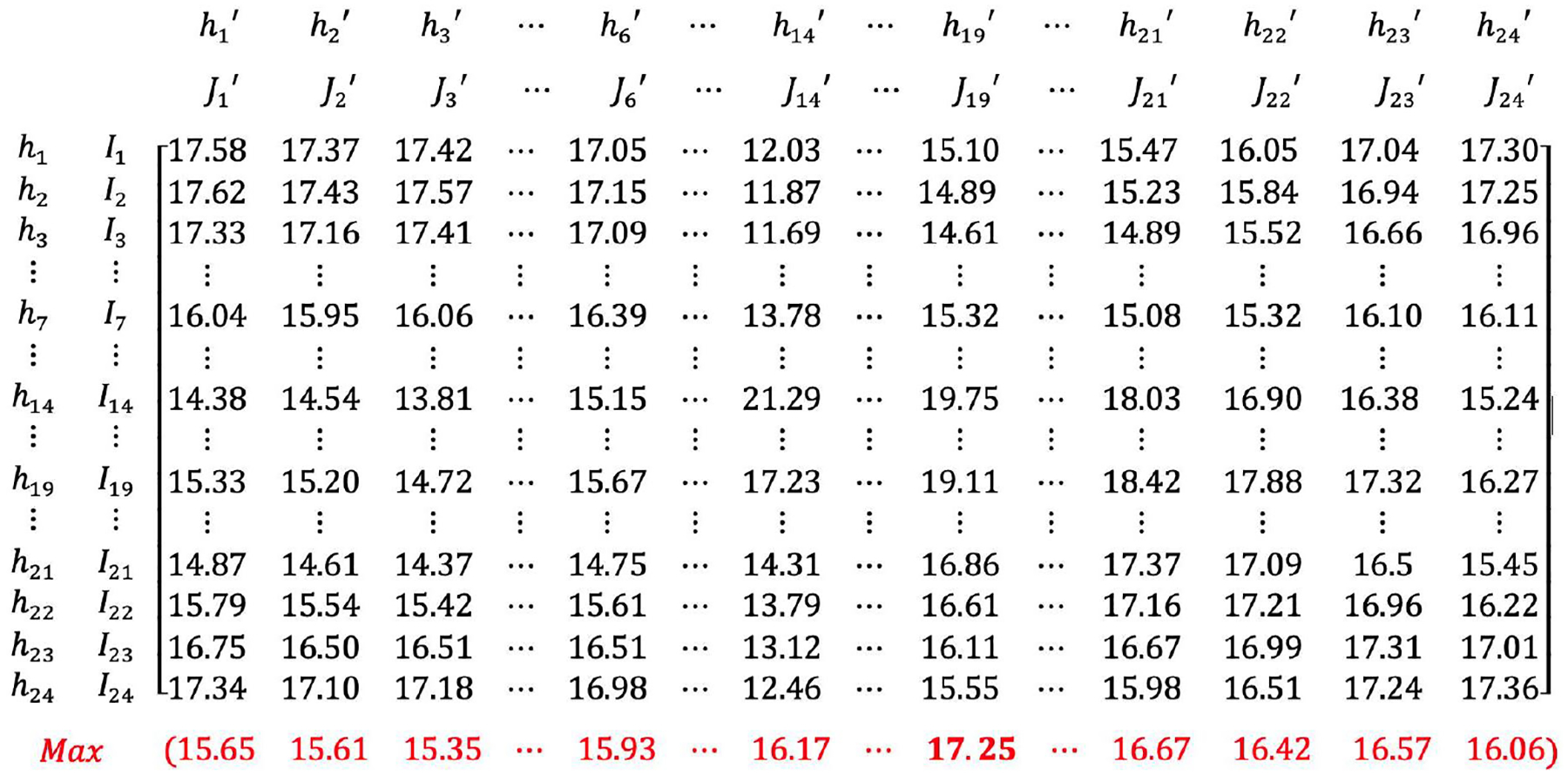
A Matrix Showing the Whole Process of the OSTO on the Case in Washington DC (k = 100). All numbers in Figure 3 are percentages. Numbers in black show the AED coverage rate when applying the solution in a specific hour (e.g., $J_{1}^{\prime }$) to cover a visitor distribution at all hours (e.g., $h_{1},h_{2},\;\ldots ,h_{24}$). Numbers in red show 24 of the average performances $\left(N_{J_{h^{\prime }}^{\prime }}\right)$ of each solution. The number marked in red bold is the highest average performance 17.25% (when $J_{19}^{\prime }$ is applied to all hours).

**Figure 4.**
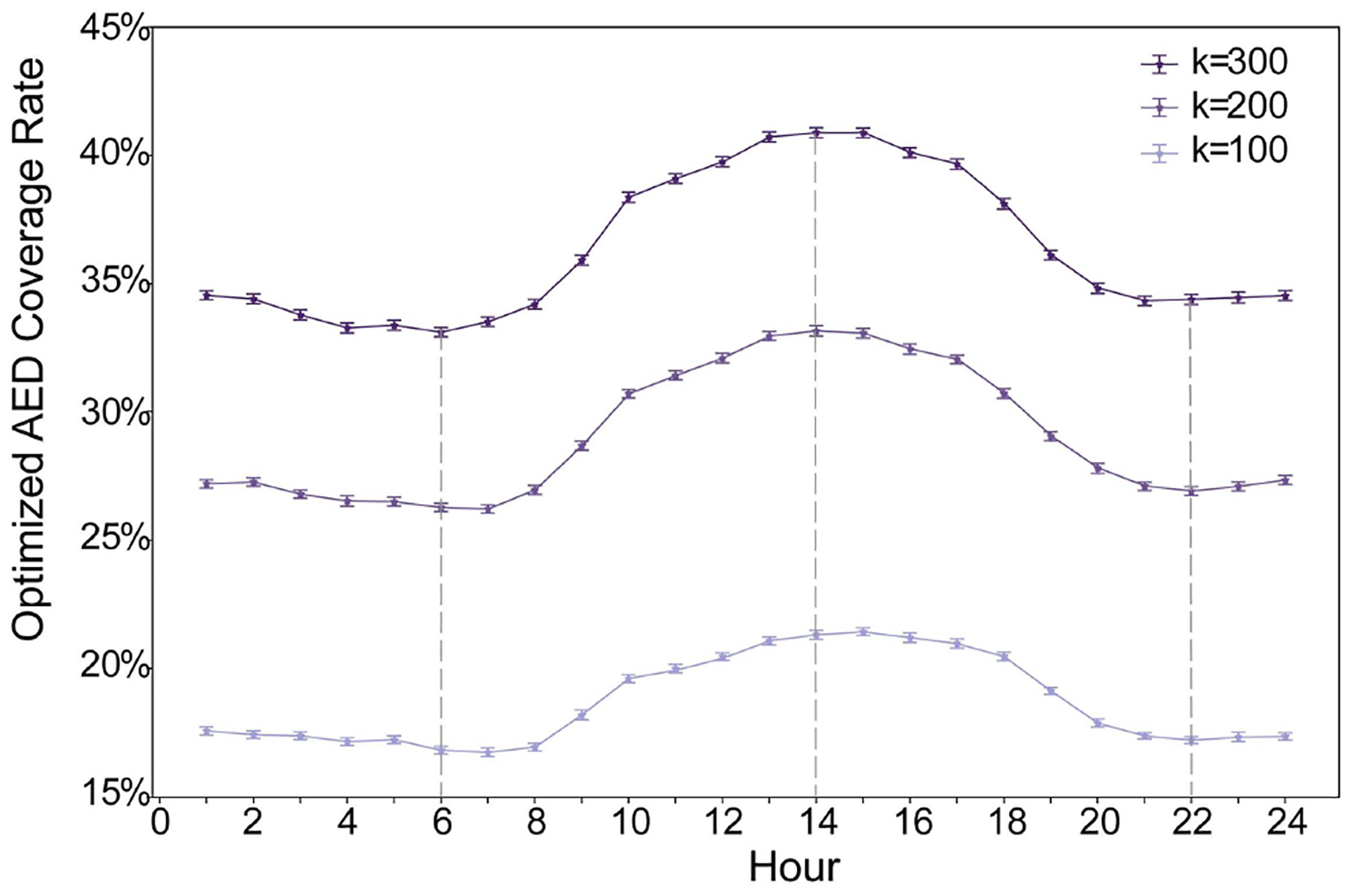
Optimized AED coverage rate. The optimized AED coverage rate $\left(R_{J_{h}^{\prime }I_{h}}\right)$ refers to the coverage rate using the set of optimized AEDs in a specific hour to cover the visitor distribution of the hour itself. For each hour, the error bar is generated from resampling 61,575 visitors from the total number of visitors 100 times.

**Figure 5.**
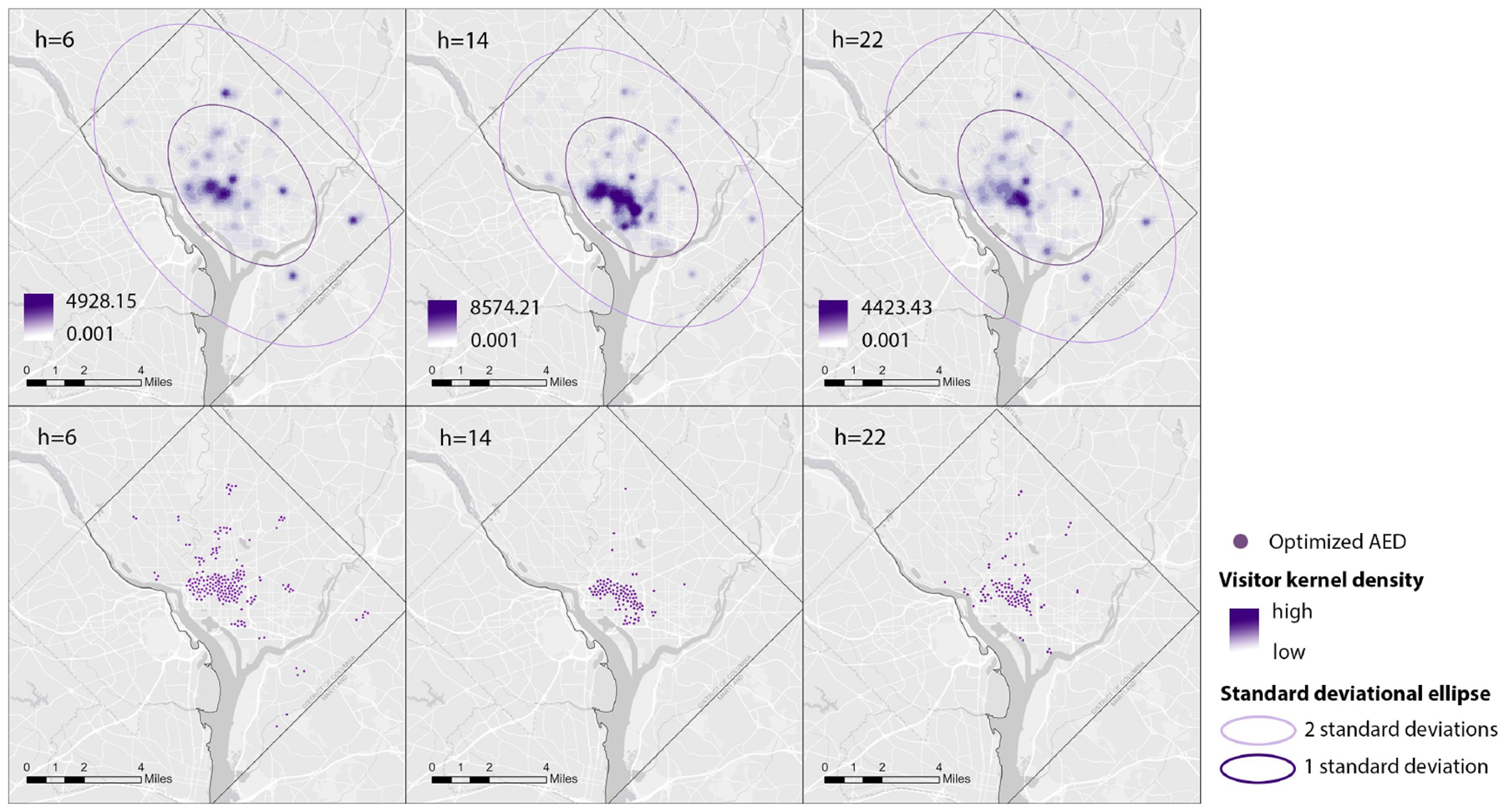
Kernel Density Maps of Visitors and Corresponding Optimized AED Locations at Three Particular Hours (k = 100).

The matrix showing the detailed result across the whole process of the OSTO in this case is illustrated in Figure 3. The results from the first stage of the OSTO are elaborated as follows. The trends of optimized AED coverage rates across 24 h a day for different ks are highly similar (Figure 4), although minor differences exist in the peak and valley hours between these three ks. In general, the peak hours for all three ks are approximately 14:00, and the valley hours are around 6:00 and 22:00, implying a general pattern of dynamic population distribution not significantly affected by the sample size k. Exploring the divergence of the optimized AED coverage rate between different hours helps us understand the regularity of how the optimization algorithm works on different visitor distributions. We analyzed the spatial clustering level of POI visitors of each hour by adopting the kernel density estimation and directional distribution analyses, and it was found that the optimized AED coverage rate is positively associated with visitor clustering level. Take three particular hours (one peak and two valleys) as an example (Figure 5). One standard deviation ellipse includes 68% of visitors, and two standard deviations ellipse includes 95% of visitors. Compared with 6:00, a more compacted directional ellipse and a higher kernel density in the central area at 14:00 indicates a higher clustering level of visitors, and a higher optimized coverage rate. Compared with 14:00, a more expanded directional ellipse and a lower kernel density at 22:00 indicate a lower clustering level and a lower optimized coverage rate. The optimized coverage rate can be referred to in Figure 4.

**Figure 6.**
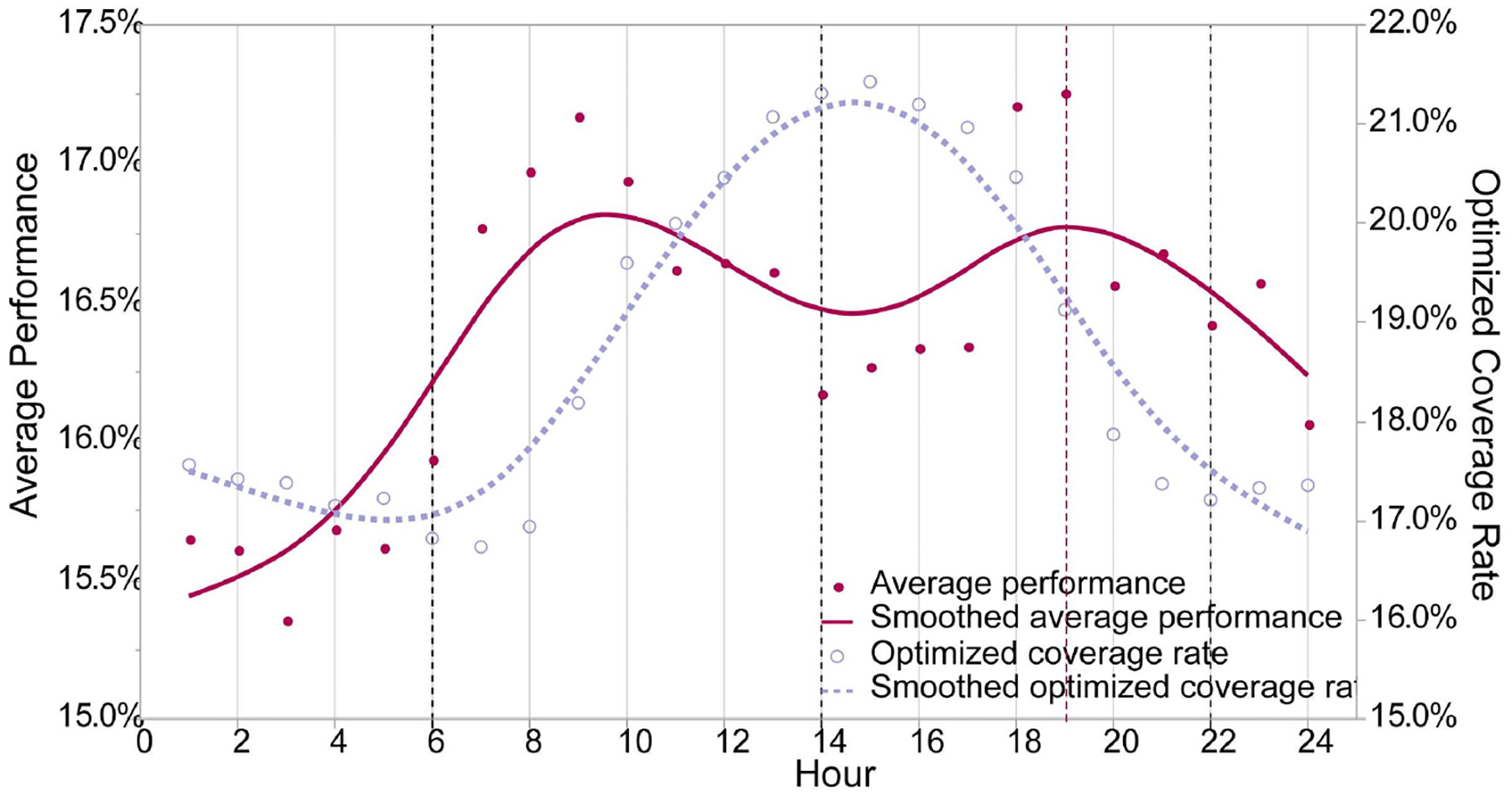
Comparison between the Average Performance of Optimized AEDs and the Optimized AED Coverage Rate across 24 h (k = 100). The average performance of each set of optimized AEDs $\left(N_{J_{h^{\prime }}^{\prime }}\right)$ refers to the average performance applying each set of optimized AEDs to 24 sets of visitor distributions. The smoothed line is calculated based on a cubic spline with a lambda of 0.05.

The results from the second stage of the OSTO are also elaborated as follows. The hour of 19:00 has the highest average performance of optimized AEDs (Figures 3 and 6). The trend and the magnitude of the average performance across 24 h were compared with that of the optimized AED coverage rate with k equals 100 (Figure 6). The curve of the optimized AED coverage rate is almost reversed to that of average performance. We noticed exceptions from the reversed shape at the beginning and end of the curves, but in general, the reversal relationship dominates. Specific values of optimized AED coverage rates and average performances at particular hours $\left\{h_{6}^{\prime },h_{14}^{\prime },h_{19}^{\prime },\;\ldots \;,h_{22}^{\prime }\right\}$ are shown in Figure 3. For the magnitude comparison, the range of the magnitude of optimized AED average performance is smaller than that of the optimized AED coverage rate. Average performance on all 24 h can help mitigate the extremely high or low optimized coverage rate caused by a particular visitor distribution.

#### 3.3.2. Relocating Existing AEDs in Washington DC

**Figure 7.**
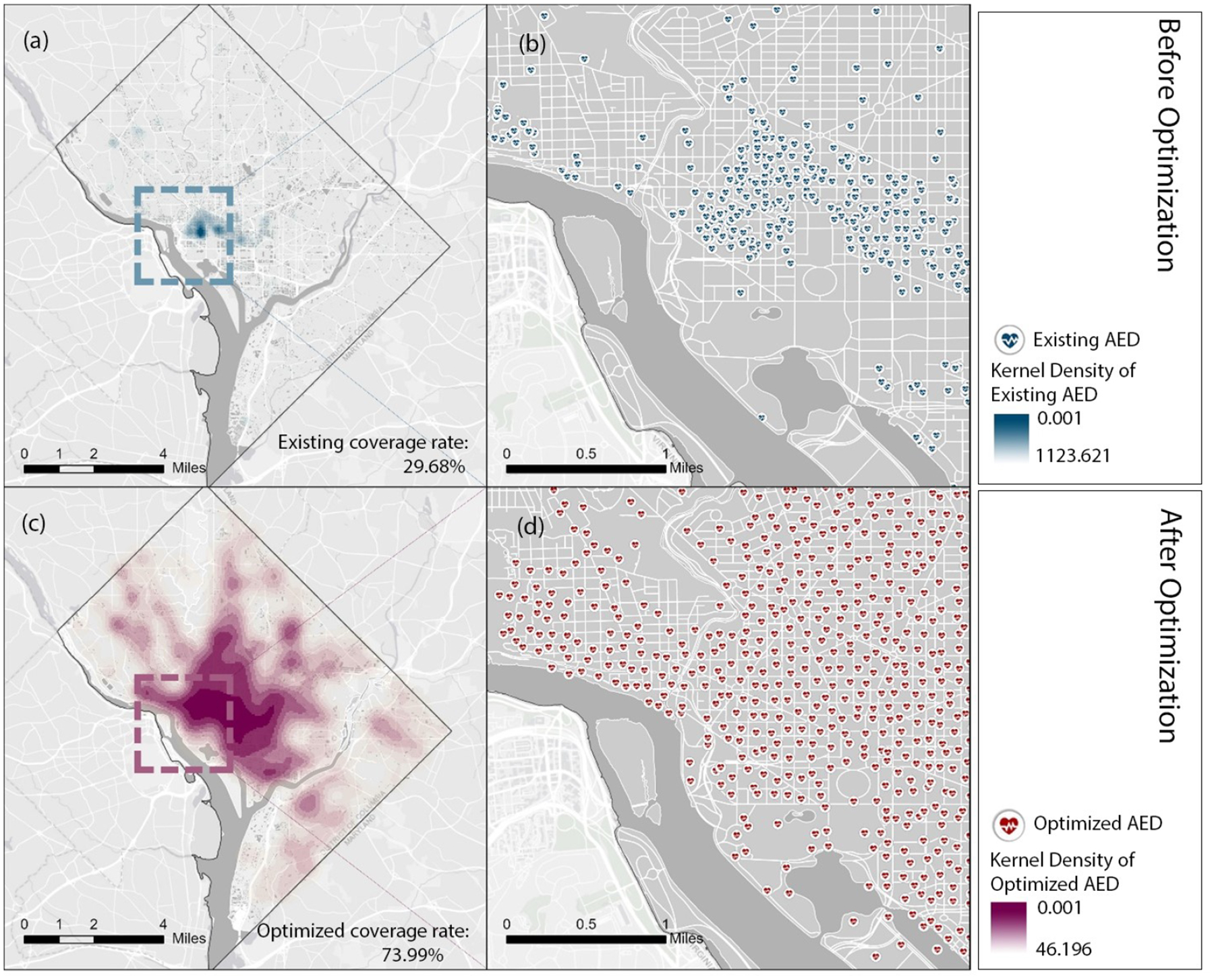
Comparison of the Distributions between Existing AEDs and Relocated AEDs in Washington DC. (**b**,**d**) shows the detailed locations of existing and relocated AEDs in a zoomed area. The kernel density maps (**a**,**c**) are computed based on the locations of existing and relocated AEDs.

To make the results before and after optimization comparable, we calculated the existing AED coverage rate based on the average performance of existing AEDs on all visitor distributions of 24 h. The unsatisfactory rate of 29.68% calls for an improvement in AED placement. Based on the result that the AED solution at 19:00 a higher performance than at any other time, we adopted the visitor distribution at 19:00 to optimize and relocate the existing AEDs. The AED coverage rate is improved to 73.99%. As shown in Figure 7, there is a great difference in AED spatial pattern before and after optimization. Before optimization, AEDs in the central area have a significantly higher density than those in peripheral areas. The range between the highest and lowest density reached 1124. After optimization, AEDs spread around, and the range between the highest and lowest densities narrowed down to 46.

#### 3.3.3. Cost–Coverage Increment Curve

**Figure 8.**
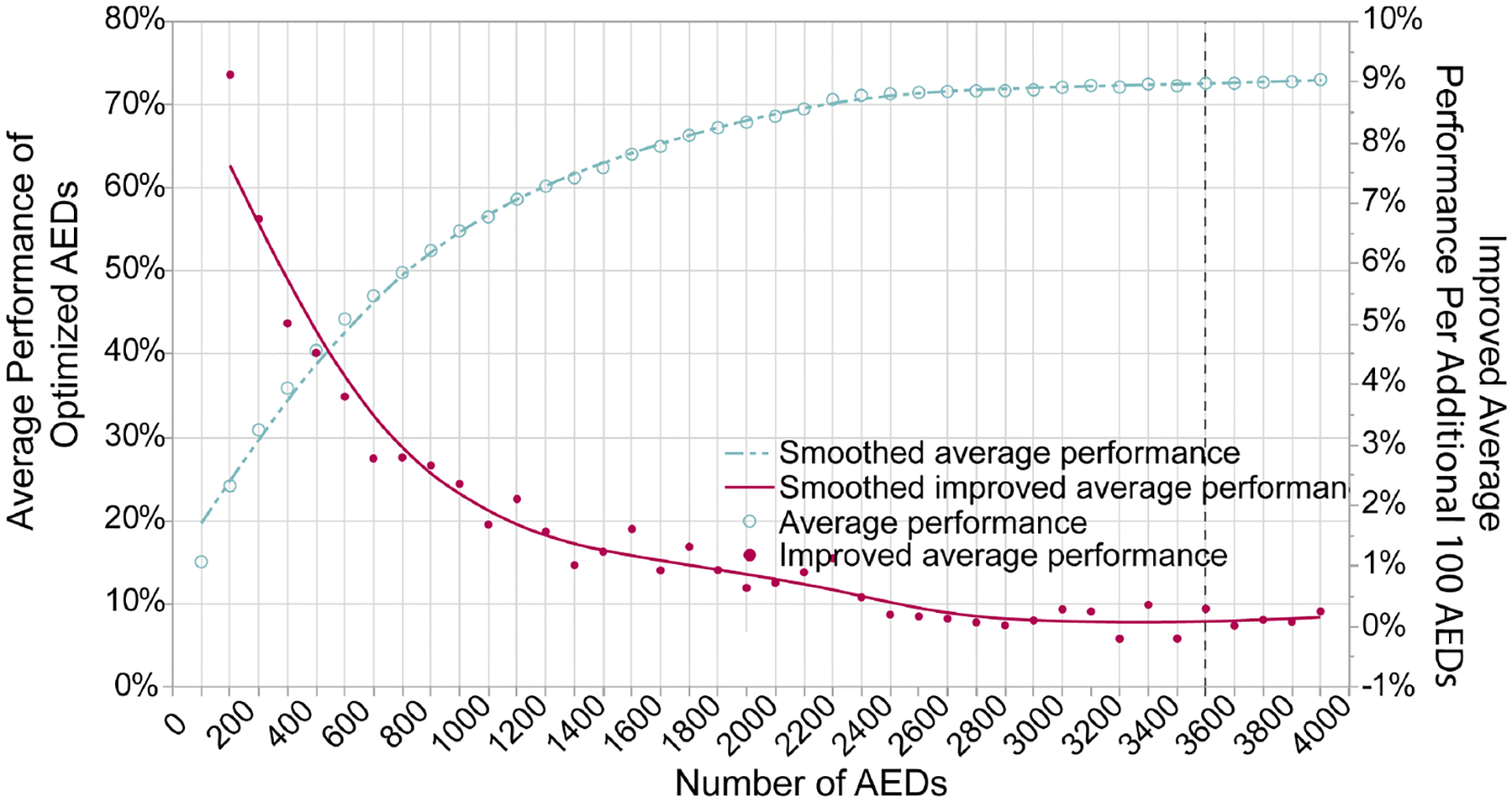
Cost–Coverage Increment Curve.

In view of the similarity of the trends of optimized AED coverage rate between different ks, it is extrapolated that, regardless of the k value, the average performance of optimized AEDs will always reach the highest at 19:00. Therefore, we computed the cost–coverage increment curve based on the visitor distribution at 19:00. From the average performance curve (Figure 8), the average performance increases with more AEDs at first. However, the plateau is reached after 3600 AEDs. From the curve of improved average performance (cost–coverage increment curve), the gain of improved performance per additional 100 AEDs is decreasing monotonically. After the threshold of 3600, the performance will only increase marginally. The improved average performance per additional 100 AEDs (Δk) is shown as $\Delta N_{J_{h^{\prime }}^{\prime }}\left(h^{\prime }=19\right)$. Both smooth lines are calculated based on a cubic spline with a lambda of 0.05.

## 4. Discussion

### 4.1. Comparison of Current Planning and Optimized Planning of AEDs

Previous spatial analytic methods, including the AHA guideline-based and population–based method, could lead to a paradoxical result with many AEDs placed in an area with a relatively low risk of OHCA. For example, the existing AED placement in Washington DC, which follows AHA guidelines, puts a disproportionate number of AEDs (1134 out of 2113) in downtown areas where the evaluated risk of potential OHCA incidence is relatively low. However, our study adopting the spatio-temporal optimization method demonstrated the improved AED coverage level. Such an increment results in shortened average time from collapse to treatment, which could positively improve the probability of survival. Compared with the existing plan in Washington DC, our proposed method deployed 298 out of 2113 AEDs downtown based on evaluating potential OHCA distribution. This deployment plan maximized the AED coverage rate across all areas at risk and balanced the supply and demand to a certain extent.

### 4.2. Designing an Inclusive Strategy Considering Potential OHCA Distributions across All SpaceTime Ranges

The optimized AED coverage rate (Figure 4) showed highly similar trends across 24 h between different numbers of AED to be configured. This result implicated that the trends of the AED coverage rate were only affected by the spatial pattern of visitors at different times. This effect may result from the MIP model that always deploys AEDs proportionally concerning the distribution of the potential risk of OHCAs when maximizing the coverage level, regardless of the number of AEDs to be configured.

We also observed a reversed relationship between the trend of the optimized AED coverage rate and the trend of average performance across 24 h (Figure 6). We attribute this effect to the correlation between the visitor clustering level and the optimized pattern of AED locations. For hours with more clustered visitors, the optimized AED locations on that hour will follow a high-density pattern. Although this can give a high coverage rate for that hour on the visitor distribution of the hour itself, the performance of the optimized AEDs on all hours’ visitor distribution will be alleviated. A high-density set of AEDs is more challenging to couple with all kinds of visitor distributions. Selecting the solution from hours with low-density visitors could improve the overall coverage level across all spaces and times. These findings should initiate critical thinking for researchers and policymakers that the overall solution in a given geographic area needs to be designed to serve all the potential risk populations at any space and time, instead of simply maximizing a population shown in a limited space–time domain.

Finally, to make our solution of optimized AEDs more inclusive of potential OHCAs in different time periods, we applied the average performance evaluation and identified the solution with the highest performance based on the original purpose of maximizing efficiency. This efficiency maximization strategy is supported by utilitarianism to maximize the sum of benefits for all people [36]. However, from another perspective of egalitarian theories, which encourages a policy on equalizing the relative level of accessibility between different groups [37], we might need to adjust the performance evaluation by selecting the solution with the smallest coverage gap. In further studies, careful consideration and exploration of the moral interpretation of utilitarianism and egalitarianism in adopting different policy measures should be conducted.

### 4.3. Limitations

There are several limitations to our study. First, this study is contingent upon the reliability and accessibility of SafeGraph data. However, in real-world situations, these data may be limited by drawbacks such as the inadequate coverage of mobile device signals, the inaccurate counting of visit data, or only encompassing public or commercial buildings, which neglects 39.68% of out-of-hospital cardiac arrests [21]. In the future, the limitations of this study could be overcome by implementing cutting-edge communication technologies such as 5G and indoor positioning systems, as well as the procurement of more comprehensive datasets. Second, we optimistically assumed that all bystanders within proximity could locate and access AEDs and that all the AEDs could be appropriately used. However, this assumption first failed to consider the lack of guidance applications to locate AEDs. For access to AED, this assumption also ignores physical obstructions such as walls or multistory buildings that may impede access to AEDs or extend the access time, leading to an overestimation of the AED coverage level. This also ignores that using a Euclidean distance to represent an actual distance may also overestimate the rate. For the use of AEDs, this assumption assumes that everyone is trained and knows how to use AEDs correctly. Combining accessibility optimization with several practical measures, including placing AEDs in visible places, developing an app or online website for checking, and publicizing AED training, can help encourage the full use of AEDs. Including vertical space factors such as elevator speed in multistory buildings and elevation in a network analysis can improve the accuracy of predicting the AED access time, assisting in designing a more precise AED deployment plan. Thirdly, limiting the temporal analysis to monthly intervals with SafeGraph data hinders the ability to differentiate between holidays, weekdays, and weekends. A more refined time unit can enable a more detailed and adaptive plan for AED placement. Fourthly, the parameter setting in the optimization algorithm, such as the accessibility distance to AEDs and the walking circle size used to simulate visitor mobility, can impact the optimization result. Finally, the age distribution of OHCA demonstrates the peaks during infancy and after the age of 45 [38]. Taking age into the spatial and temporal optimization model may improve the accuracy of locating AEDs.

## 5. Conclusions

Using the OSTO method, our model provides an improved AED deployment solution under specific conditions as well as multiple options for a decision maker to quantify the trade-off between the budget and coverage level. Compared to the previous literature, where the instantaneous characteristics and critical time relevance of OHCA occurrences are often ignored, our results remind both researchers and policymakers that healthcare facilities’ placement schemes should reasonably consider all populations across time and space. In addition, our cost-coverage increment analysis revealed that solely increasing the number of healthcare facilities may not guarantee an increasing coverage rate. The trade-off between the budget and coverage level need to be balanced in real scenarios. Furthermore, in addition to the AED deployment optimization planning shown in this study, our model is highly transferable to other scenarios for tackling different healthcare facilities’ deployment tasks with certain constraints. Nevertheless, considering the current limitations illustrated in the previous section, this method can be improved, but it also takes us one step closer to applying this approach in a real scenario.

## Data Availability

Data available upon request due to restrictions, e.g., privacy or ethical.
